# Sleep Status and the Associated Factors: A Large Cross-Sectional Study in Shaanxi Province, China

**DOI:** 10.3390/ijerph18031250

**Published:** 2021-01-30

**Authors:** Yaxuan Zhang, Jiwei Wang, Xinyuan Lu, Beibei Che, Jinming Yu

**Affiliations:** School of Public Health, Fudan University, Shanghai 200032, China; joycezhangyx@163.com (Y.Z.); jiweiwang@fudan.edu.cn (J.W.); 18211020007@fudan.edu.cn (X.L.); 18211020047@fudan.edu.cn (B.C.)

**Keywords:** sleep quality, sleep problems, associated factors

## Abstract

This study aimed at investigating the sleep status and its associated factors in Shaanxi province, China. We conducted a cross-sectional study among 11,399 subjects in Shaanxi Province, China. Data were collected via spot field questionnaire surveys. The contents included demographic characteristics, sleep status, lifestyles, disease history and other associated factors. Logistic regression analysis was used to estimate the effect of associated factors on sleep quality. A total of 11,036 subjects were included in the final analysis. In total, 12.8% of the participants had bad or very bad sleep. In the last month, 8.4% of the participants had difficulty in initiating sleep, 7.6% of the participants had difficulty in maintaining sleep, 8.8% of the participants suffered from awakening earlier and 10.3% of the participants had the problem of feeling sleepy during the day ≥3 times per week. Poorer sleep quality was associated with being female, being unmarried or without cohabiting with a boyfriend/girlfriend, being divorced or widowed, heart diseases, musculoskeletal diseases, concerns about their own health, drinking alcohol, taking hypnotics, and a longer daily screen time. Better sleep quality was associated with medium education level, high family monthly income, good self-reported health status, and having breakfast regularly. In conclusion, more than one in ten people did not sleep well and suffered from different sleep problems in Shaanxi, China. Sleep quality was associated with sex, marital status, educational level, family monthly income, heart disease, musculoskeletal diseases, degree of concerning about their own health, self-reported health status, drinking alcohol, having breakfast, taking hypnotics and daily screen time.

## 1. Introduction

Sleep disturbance is becoming a more and more important health issue around the world. Sleep disorders may influence people’s daily life and give rise to many health problems. Sleep deprivation will affect humans’ physical and cognitive performance [1]. Sleep apnea may cause a batch of cardiovascular consequences, such as hypertension, coronary heart disease, arrhythmia, heart failure, and stroke [2]. Lack of sleep may have a negative impact on weight management [3]. Impaired sleep is both a risk factor and a symptom of depression [4]. Appropriate sleep duration has a protective effect on type 2 diabetes compared with short or long sleep duration [5].

With the development of China, people face fierce competition and suffer from great life stress, which increases people’s anxiety. Anxiety symptoms are associated with sleep problems [6]. Nevertheless, few studies have focused on people’s sleep quality and its associated factors in China.

Several previous studies investigated the sleep quality of students [7,8]. A cross-sectional study among students in Macao, China showed that irregular bedtime was associated with elevated body weight [7]. Another cross-sectional survey among Inner Mongolia Medical University students found that exercise less than three times a week and skipping breakfast were associated with poor sleep quality [8]. Some researchers investigated sleep quality and its associated factors among rural adults [9,10], low-income rural adults [11], and rural elderly [12,13]. A cross-sectional survey conducted in Deqing County, China reported that rural people who were unmarried or had low personal income or any chronic diseases were more likely to have poor sleep quality [9]. Another study among rural adults in Henan province, China showed that older age, being female, being unmarried/divorced/widowed, low education level, low income, drinking, depression and dyslipidemia could increase the odds of poor sleep quality [10]. A cross-sectional survey among low-income adults in a rural area of China found that older age, unemployment, lower income, disability and chronic disease comorbidities were associated with poor sleep quality [11]. Two cross-sectional studies among rural elderly in China showed that cardiometabolic risk factors and disorders were associated with poor sleep quality [12]; chronic disease, advanced age, low quantities of staple food, rice as major food, poor Physical Component Summary, poor Mental Component Summary, and significant dysfunction of Activities of Daily Living were predictors for poor sleep quality [13].

In this case, our research investigated sleep quality and its associated factors among adults from urban and rural areas on a large scale in Shaanxi Province, China.

## 2. Materials and Methods

### 2.1. Study Site and Population

This cross-sectional study was conducted in Shaanxi Province, China. Seven cities and one demonstration zone were involved in this study. They were Yan’an city, Yulin city, Ankang city, Hanzhong city, Xi’an city, Weinan city, Baoji city and Yangling Demonstration Zone. Participants aged from 18 to 90 who could read and complete the questionnaire were recruited.

### 2.2. Investigation Methods

We extracted one district and one county from each city, which ensured that urban populations and rural populations were both investigated. Subjects were conveniently sampled as clusters from urban or rural communities in May to June 2019. Spot field questionnaire surveys were conducted by trained investigators. Data were collected anonymously.

The study was conducted in accordance with the Helsinki declaration and approved by the Ethics Committee for Medical Research, School of Public Health, Fudan University. (IRB00002408, FWA00002399; approval number IRB#2019-04-0741) Written informed consent was obtained from all participants or their legal guardians before investigation. The questionnaire used in our study was developed for this study.

The questionnaire contained sociodemographic characteristics, lifestyles, health status, disease history and self-reported sleep quality. Sociodemographic characteristics included sex, age, marital status, educational level, personal monthly income, and family monthly income. Body mass index (BMI) is calculated by weight(kg)/height2(m2) and categorized into 4 groups [14].

Lifestyle factors included physical activities, smoking, drinking alcohol, having breakfast, eating vegetables, eating fruits, square dancing, daily screen time, using electronic devices before sleep, and time of exposure to sunlight. Physical activity was measured by average times of at least 30 minutes of physical activity (physical activity of moderate intensity or above, such as brisk walking, running, swimming, dancing, cardio, football, basketball, climbing, etc.) per week. Smoking was measured by average number of cigarettes smoked each day. Drinking alcohol was measured by frequency of drinking. Having breakfast was measured by average times of having breakfast per week. Eating vegetables was measured by average weight of vegetables eaten each day, which excluded starchy vegetables such as potato, yam, taro, etc. Eating fruits was measured by average times of eating fresh fruits per week, which excluded canned fruit, preserved fruit, etc. Square dancing was defined as clustered dance in outdoor places such as parks in China and measured by average times per week. Daily screen time was measured by average time of daily use of electronic devices such as cellphones, televisions and computers. Using electronic devices before sleep was defined as using electronic devices between bedtime and 30 minutes before bedtime, and measured by how often. Time of exposure to sunlight was measured by daily average sunlight exposure time (minutes) in the last month.

Subjects were asked the degree of concerning about their own health and self-reported health status.

We also measured whether they were suffering from chronic noncommunicable diseases which were diagnosed by a doctor. Chronic diseases in our study included hypertension, diabetes, hyperlipidemia, heart disease (such as coronary heart disease, rheumatic heart disease, cardiomyopathy, congenital heart disease, pulmonary heart disease and so on), respiratory diseases (such as impaired lung function, chronic obstructive pulmonary disease, asthma and so on), stroke (such as transient ischemic attack, ischemic stroke, hemorrhagic stroke and so on), malignant tumors, hyperuricemia, and musculoskeletal diseases (such as osteoarthritis, rheumatoid arthritis and so on).

### 2.3. Measurement of Sleep Status

Self-reported sleep quality (hereinafter referred to as sleep quality) was measured with a 5-ordinal-categorized answer, which means that answers 1 to 5 correspond with very good, good, general, bad and very bad, respectively.

According to the diagnosis of insomnia in the Diagnostic and Statistical Manual of Mental Disorders, Fifth Edition (DSM-V) [15], participants were asked the following questions to obtain more sleep information:

(i) In the last month, the frequency of difficulty initiating sleep for at least 30 min;
(ii) In the last month, the frequency of difficulty maintaining sleep, waking more than 3 times in a night;
(iii) In the last month, the frequency of awakening earlier than usual in the morning, at least 30 min early, with an inability to return to sleep;
(iv) In the last month, the frequency of feeling sleepy during the day;
(v) How long have you had one of the above sleep problems (difficulty initiating sleep, difficulty maintaining sleep, awakening early)?
(vi) In the last month, have you taken medication (e.g., hypnotics such as benzodiazepines or sedatives) to help you sleep?

### 2.4. Statistical Analysis

Data entry was completed by Epidata 3.1 (EpiData Association, Odense, Denmark) and statistical analysis was performed by SAS 9.4 (Copyright by SAS Institute Inc., Cary, NC, USA. Licensed to FUDAN UNIVERSITY). The significance level was set at 0.05. Continuous variables were described as mean and standard deviation (SD), and categorical variables were described as frequencies and percentage (%).

Logistic regression analysis was used to estimate the effect of influencing factors on sleep quality, and crude odds ratio (cOR) and adjusted odds ratio (aOR) and their 95% confident intervals (CI) were calculated. Self-reported sleep quality was categorized as good sleep quality (*y* = 0) and bad sleep quality (*y* = 1). Answers 1 to 3 (very good, good, general) were categorized as good sleep quality, and answers 4 and 5 (bad and very bad) were categorized as poor sleep quality. The variables including personal monthly income, family monthly income, degree of concerning about their own health, self-reported health status, taking medication to help sleep, physical activities, smoking, drinking alcohol, having breakfast, eating vegetables, eating fruits, square dancing, daily screen time and using electronic devices before sleep were treated as continuous variables in logistic regression. The stepwise method was used for multivariate regression.

## 3. Results

### 3.1. Subjects Characteristics and cOR

A total of 11,036 subjects were included in the final analysis, and 363 (3.2%) subjects were excluded due to missing data or invalid questionnaires. Subjects’ characteristics and cOR are described in Table 1 grouped by sleep quality. Our study included participants aged from 18 to 90 and with a total average of (46.8 ± 15.57) years. There were 35.4% males and 59.2% females. Most men (89.3%) and women (85.8%) had good sleep. There were 58.6% of participants with a normal BMI. Most (72.7%) were married or remarried or cohabiting with a boyfriend/girlfriend. Around one third of them were highly educated. Only 13.9% of participants reported a personal monthly income RMB ≥ 4000, and 21.8% of participants reported a family monthly income RMB ≥ 8000. The time of exposure to sunlight was (164.5 ± 128.14) minutes. Most participants were concerned about their own health, and the majority of them reported a good health status. Only 14.3% of participants had taken medication (e.g., hypnotics such as benzodiazepines or sedatives) to help them sleep in the last month. Many of them exercised more than once per week. Three quarters were non-smokers, and 68.8% of all participants were non-drinkers. More than half had breakfast every day. Almost 60% of participants ate at most 200g of vegetables each day. Nearly half (46.4%) of participants ate fruits no more than twice per week. Almost one quarter (23.5%) went square dancing from time to time (occasionally or ≥1 time per week). About one third (32.8%) used electronic devices more than 3 hours per day, and 44.5% of participants frequently (often, almost every day) used electronic devices before sleep. There were 17.6% of participants with hypertension, 6.0% with diabetes, 8.1% with hyperlipidemia, 6.8% with heart disease, 2.4% with respiratory diseases, 1.6% with stroke, 0.4% with malignant tumors, 0.6% with hyperuricemia and 4.3% with musculoskeletal diseases.

The cOR is shown in the table below. Poorer sleep quality was associated with older age (*p* < 0.001), being female (*p* < 0.001), BMI < 18.5 (*p* = 0.038), being divorced or widowed (*p* < 0.001), primary school or below educational level (*p* < 0.001), taking hypnotics (*p* < 0.001), smoking (*p* = 0.012), frequently using electronic devices before sleep (*p* = 0.038), hypertension (*p* < 0.001), diabetes (*p* < 0.001), hyperlipidemia (*p* < 0.001), heart disease (*p* < 0.001), respiratory diseases (*p* < 0.001), stroke (*p* < 0.001), malignant tumors (*p* = 0.009), hyperuricemia (*p* = 0.028), and musculoskeletal diseases (*p* < 0.001), while better sleep was associated with high personal monthly income (*p* < 0.001), high family monthly income (*p* < 0.001), concerning about their own health (*p* = 0.007), good self-reported health status (*p* < 0.001), having breakfast regularly (*p* = 0.022), eating more vegetables (*p* = 0.018), and eating more fruits (*p* < 0.001).

### 3.2. Sleep Status

In this study, 50.7% of participants had good or very good sleep, 36.5% of participants’ self-reported sleep quality was general, and 12.8% of participants had bad or very bad sleep. In the last month, 8.4% of participants had difficulty in initiating sleep ≥3 times per week, 7.6% of participants had difficulty in maintaining sleep ≥3 times per week, 8.8% of participants suffered from awakening earlier ≥3 times per week, and 10.3% of participants had the problem of feeling sleepy during the day ≥3 times per week. In total, 83.5% of participants had one of the above sleep problems (difficulty initiating sleep, difficulty maintaining sleep, awakening early) ≤3 months, and 16.5% of participants had one of the above sleep problems >3 months. The detailed information of sleep status is described in Table 2.

### 3.3. The Associated Factors of Sleep Quality

The associated factors obtained by stepwise regression analysis are shown in Table 3. In our study, female participants slept poorer than male participants (aOR = 1.792, *p* < 0.001). Subjects with a life partner slept better than those without a life partner, the aOR of unmarried participants or those not cohabiting with a boyfriend/girlfriend was 1.319 (95% CI = 1.022–1.704), and the aOR of divorced or widowed participants was 1.436 (95% CI = 1.052–1.960). The highly educated (college and above) participants slept worse than those less educated (middle/high school) (aOR = 0.748, *p* = 0.003). Participants slept better with increased family monthly income (aOR = 0.939, *p* = 0.004). Subjects with chronic noncommunicable diseases such as heart disease (aOR = 1.478, *p* = 0.002) and musculoskeletal diseases (aOR = 1.460, *p* = 0.018) had poorer sleep. Participants slept poorer if they were more concerned about their own health (aOR = 1.113, *p* = 0.016), but they slept better if they reported a better health status (aOR = 0.490, *p* < 0.001). With the increase in frequency of drinking alcohol (aOR = 1.201, *p* < 0.001), subjects slept poorer. Participants eating breakfast regularly (aOR = 0.935, *p* = 0.016) had better sleep. Subjects who took hypnotics more frequently (aOR = 1.643, *p* < 0.001) had poorer sleep. People had poorer sleep if they had a longer daily screen time (aOR = 1.055, *p* < 0.001).

## 4. Discussion

In our study, self-reported sleep quality was generally poor in the population aged from 18 to 90 of Shaanxi, China.

From this study, we found that sleep quality was associated with sex, marital status, educational level, family monthly income, heart disease, musculoskeletal diseases, degree of concerning about their own health, self-reported health status, drinking alcohol, having breakfast, taking medication to help sleep and daily screen time.

In our study, female participants slept poorly compared with male participants. A descriptive, cross-sectional study conducted in Korea reported a consistent conclusion that being female was a significant risk factor for poor sleep quality [16]. This may be because women tend to experience a greater prevalence of anxiety disorders [17]. Participants who had a life partner slept better than those without a life partner. The reason may be that a life partner can provide emotional support to help them sleep well. From another point, there might be bidirectional relationships between marital status and sleep problems, which was supported by a 4-year follow-up study in a Korean cohort. This study reported that poor marital quality is a risk factor for sleep disturbance for older adults, and on the other hand, sleep disturbance may have a negative impact on marital quality for all age groups [18]. The same finding was also reported by a study which suggested that people in the older population with widowed, divorced or separated marital status were more likely to have sleep complaints [19]. This result was consistent with our study. Subjects with a medium education level slept better than highly educated and less educated subjects. The possible reason is that the highly educated population may have higher work stress (such as more intense competition in daily work), while less educated people may face higher survival stress (such as lower salary). Our study suggested that with the increase in family monthly income, participants sleep better. However, personal monthly income was not a significant factor, which is probably because family members always live together in China. According to the literature, a longitudinal community study named Ibadan Study of Ageing found that decreasing economic status had an association with increasing incidence of insomnia [20]. The results of our study indicated that participants with chronic noncommunicable diseases such as heart disease and musculoskeletal diseases have poor sleep. A prospective cohort study found that both short sleep duration and poor sleep quality increase the risk of coronary heart disease [21]. Furthermore, a meta-analysis of prospective cohort studies further supported this result as this research reported that sleeping less than 7 hours or more than 8 hours are both associated with a higher risk of mortality and cardiovascular events, and longer sleep may have a strong association with adverse outcomes compared with shorter sleep [22]. In another way, heart disease may affect sleep quality through biological mechanisms, which could be supported by a cross-sectional study from the Maintenance of Balance, Independent Living, Intellect, and Zest in the Elderly Boston Study, which found that musculoskeletal pain was significantly associated with difficulties in initiating sleep, maintaining sleep or sleeping longer than usual in older adults [23]. Participants slept poorer when they were more concerned about their own health, but slept better if they reported a better health status. One reason may be that healthy people have better sleep and they are less concerned about their own health. With the increase in frequency of drinking alcohol, participants had poorer sleep. A study in the greater Los Angeles area recruited a number of non-treatment-seeking problem drinkers and showed that alcohol problem severity has a significant association with sleep disturbance [24]. Participants who had breakfast regularly had better sleep. According to the literature, a cross-sectional study from the Project Eating and Activity in Teens and Young Adults study found that poor sleep quality was associated with breakfast skipping and other problematic eating behaviors [25]. Another study supported this view that breakfast consumption may improve perceived sleep quality compared with breakfast skipping in healthy young adults [26]. One possible reason may be Night Eating Syndrome because night eating may have a negative effect on sleep quality and decrease appetite in the morning. Furthermore, we found that people had poorer sleep associated with a longer daily screen time. This point could be supported by a cross-sectional study that reported a similar result that over use of mobile cellphones is associated with poor sleep quality and quantity [27]. There are some possible mechanisms as follows. The first is that people take sleep time to use electronic devices [28]. Second, psychological and physical arousal due to the content of the media and social interaction may also interfere with the ability to fall and stay asleep [28]. Finally, there is the effect of light on both circadian rhythm and alertness [28].

In accordance with the present results, a previous study that investigated participants’ sleep quality in Hunan Province, China demonstrated that being female, older age, higher education level, being unmarried, smoking and drinking alcohol were associated factors of insomnia [29]. The results of our study also showed that being female, being unmarried and drinking alcohol were associated with poor sleep quality, but we did not find an association between age/smoking and sleep. Another study that researched the sleep quality of people in Liaoning Province, China reported that 11.59% of the population had sleep quality problems, and the sleep quality problems of women, people over 40, divorced people and poor people were more prominent [30]. These results were similar to our results that 12.8% of the participants had bad or very bad sleep, and being female, being divorced and higher family income were associated factors of poor sleep quality. The above studies only surveyed in one province, but there was a previous study that investigated the sleep status of adults aged 18–64 in 15 provinces of China in 2015 [31]. This study further demonstrated that subjects in cities and western regions of China had shorter sleep duration.

This study suggested that many people in Shaanxi Province, China had poor sleep quality, which was associated with several factors. The local government should pay more attention to sleep vulnerable populations such as females, divorced people, people with low family income, people with heart disease or musculoskeletal diseases, and people taking hypnotics to help them sleep. Meanwhile, behaviors associated with poor sleep quality, such as drinking alcohol, erratically eating breakfast, and prolonged daily screen time, should be brought into focus.

There are some limitations in our study. This is a cross-sectional study, so causal inference was limited. Besides that, we used self-reported sleep quality rather than objective indicators and we only used sleep quality as an outcome variable, which did not integrate the information of sleep problems. In further research, we will try to explore and find a proper method to integrate the six questions about sleep status into one index for model building. In addition, previous research has shown that there was a significant interaction between marital quality and age when studying their relationship to sleep [18]. However, we were not considering this interaction or any other interactions in our analysis. Additionally, this study did not investigate participants’ mental illness status, medication use that may inhibit sleep, and intake of coffee, tea or energy drinks, which would also be associated with sleep quality. Future studies will take these factors into consideration.

## 5. Conclusions

In this study, we found that more than one in ten people reported bad or very bad sleep and suffered from different sleep problems such as difficulty in initiating sleep, difficulty in maintaining sleep, awakening earlier and feeling sleepy during the day in Shaanxi, China. Furthermore, sleep quality was associated with sex, marital status, educational level, family monthly income, heart disease, musculoskeletal diseases, degree of concerning about their own health, self-reported health status, drinking alcohol, having breakfast, taking hypnotics and daily screen time.

## Figures and Tables

**Table 1:** Characteristics of Participants and crude odds ratio (cOR).

	Sleep Quality			cOR	*p* Value
	Good (*N* = 9628)	Poor (*N* = 1408)	Total (*N* = 11,036)		
Age (years), (mean ± SD)	46.4 ± 15.43	49.5 ± 16.27	46.8 ± 15.57	1.013	<0.001 ***
Sex, n (%)					
Male	3488 (89.3)	419 (10.7)	3907 (35.4)	1.000	reference
Female	5610 (85.8)	928 (14.2)	6538 (59.2)	1.377	<0.001 ***
Missing	530 (89.7)	61 (10.3)	591 (5.4)		
BMI, n (%)					
BMI < 18.5	634 (85.1)	111 (14.9)	745 (6.8)	1.255	0.038 *
18.5 ≤ BMI ≤ 23.9	5679 (87.8)	792 (12.2)	6471 (58.6)	1.000	reference
23.9 < BMI ≤ 27.9	2630 (86.8)	399 (13.2)	3029 (27.4)	1.088	0.201
BMI > 27.9	536 (85.5)	91 (14.5)	627 (5.7)	1.217	0.100
Missing	149 (90.9)	15 (9.2)	164 (1.5)		
Marital status, n (%)					
Unmarried and without cohabiting with boyfriend/girlfriend	753 (86.4)	118 (13.6)	871 (7.9)	1.110	0.320
Married or remarried or cohabiting with boyfriend/girlfriend	7032 (87.6)	993 (12.4)	8025 (72.7)	1.000	reference
Divorced or widowed	310 (77.9)	88 (22.1)	398 (3.6)	2.010	<0.001 ***
Missing	1533 (88.0)	209 (12.0)	1742 (15.8)		
Educational level, *n* (%)					
Primary school and below	1809 (82.4)	387 (17.6)	2196 (19.9)	1.560	<0.001 ***
Middle/high school	4524 (88.7)	575 (11.3)	5099 (46.2)	0.927	0.259
College and above	3186 (87.9)	437 (12.1)	3623 (32.8)	1.000	reference
Missing	109 (92.4)	9 (7.6)	118 (1.1)		
Personal monthly income, *n* (%)				0.896	<0.001 ***
RMB 0	2073 (83.7)	404 (16.3)	2477 (22.4)		
RMB 1–999	1001 (83.1)	203 (16.9)	1204 (10.9)		
RMB 1000–1999	1808 (87.9)	248 (12.1)	2056 (18.6)		
RMB 2000–2999	1985 (90.6)	205 (9.4)	2190 (19.8)		
RMB 3000–3999	1272 (88.7)	162 (11.3)	1434 (13.0)		
RMB 4000–4999	793 (88.6)	102 (11.4)	895 (8.1)		
RMB 5000–5999	311 (89.1)	38 (10.9)	349 (3.2)		
RMB 6000–6999	130 (90.3)	14 (9.7)	144 (1.3)		
RMB ≥7000	132 (91.7)	12 (8.3)	144 (1.3)		
Missing	123 (86)	20 (14)	143 (1.3)		
Family monthly income, n (%)				0.896	<0.001 ***
RMB 0	700 (83.0)	143 (17.0)	843 (7.6)		
RMB 1–1999	1123 (83.3)	225 (16.7)	1348 (12.2)		
RMB 2000–3999	2006 (85.5)	339 (14.5)	2345 (21.2)		
RMB 4000–5999	1976 (89.1)	242 (10.9)	2218 (20.1)		
RMB 6000–7999	1297 (88.7)	165 (11.3)	1462 (13.2)		
RMB 8000–9999	859 (88.8)	108 (11.2)	967 (8.8)		
RMB 10,000–11,999	634 (90.1)	70 (9.9)	704 (6.4)		
RMB 12,000–13,999	308 (90.9)	31 (9.1)	339 (3.1)		
RMB ≥14,000	358 (91.1)	35 (8.9)	393 (3.6)		
Missing	367 (88)	50 (12)	417 (3.8)		
Time of exposure to sunlight (minutes), (mean ± SD)	165.1 ± 128.05	160.3 ± 128.73	164.5 ± 128.14	1.000	0.212
Missing, *n* (%)	748 (84.2)	140 (15.8)	888 (8.0)		
Degree of concerning about their own health, *n* (%)				0.915	0.007 *
Very unconcerned	76 (80.0)	19 (20.0)	95 (0.9)		
Unconcerned	254 (80.9)	60 (19.1)	314 (2.9)		
General	2373 (87.2)	347 (12.8)	2720 (24.6)		
Concerned	3874 (87.4)	561 (12.6)	4435 (40.2)		
Very concerned	2952 (87.9)	407 (12.1)	3359 (30.4)		
Missing	99 (87.6)	14 (12.4)	113 (1.0)		
Self-reported health status, *n* (%)				0.459	<0.001 ***
Very bad	66 (53.2)	58 (46.8)	124 (1.1)		
Bad	447 (64.4)	247 (35.6)	694 (6.3)		
General	3448 (84.3)	641 (15.7)	4089 (37.0)		
Good	4196 (91.7)	379 (8.3)	4575 (41.5)		
Very good	1366 (94.6)	78 (5.4)	1444 (13.1)		
Missing	105 (95.5)	5 (4.6)	110 (1.0)		
Take medication to help sleep, *n* (%)				1.849	<0.001 ***
Never	8316 (89.7)	956 (10.3)	9272 (84.0)		
Rarely	715 (78.8)	192 (21.2)	907 (8.2)		
Sometimes	274 (70.4)	115 (29.6)	389 (3.5)		
Often	121 (56.3)	94 (43.7)	215 (2.0)		
Always	36 (59.0)	25 (41.0)	61 (0.6)		
Missing	166 (86.5)	26 (13.5)	192 (1.7)		
Physical activities, *n* (%)				1.009	0.761
0 times per week	2075 (86.0)	338 (14.0)	2413 (21.9)		
1–2 times per week	3807 (88.6)	491 (11.4)	4298 (38.9)		
3–4 times per week	2065 (87.1)	305 (12.9)	2370 (21.5)		
≥5 times per week	1562 (86.2)	249 (13.8)	1811 (16.4)		
Missing	119 (82.6)	25 (17.4)	144 (1.3)		
Smoking, n (%)				1.077	0.012 *
Non-smokers	7260 (87.3)	1056 (12.7)	8316 (75.3)		
Occasional smokers	972 (90.9)	97 (9.1)	1069 (9.7)		
1–10 cigarettes per day	685 (84.4)	127 (15.6)	812 (7.4)		
11–20 cigarettes per day	375 (84.8)	67 (15.2)	442 (4.0)		
>20 cigarettes per day	164 (82.0)	36 (18.0)	200 (1.8)		
Missing	172 (87.3)	25 (12.7)	197 (1.8)		
Drinking alcohol, *n* (%)				1.045	0.160
Non-drinkers	6659 (87.7)	937 (12.3)	7596 (68.8)		
Less than 1 time per month	1314 (84.8)	236 (15.2)	1550 (14.0)		
1–3 times per month	1260 (89.1)	154 (10.9)	1414 (12.8)		
1–3 times per week	266 (83.9)	51 (16.1)	317 (2.9)		
≥4 times per week	88 (81.5)	20 (18.5)	108 (1.0)		
Missing	41 (80.4)	10 (19.6)	51 (0.5)		
Having breakfast, *n* (%)				0.953	0.022 *
0 times per week	770 (85.4)	132 (14.6)	902 (8.2)		
1–2 times per week	892 (87.1)	132 (12.9)	1024 (9.3)		
3–4 times per week	1188 (85.4)	203 (14.6)	1391 (12.6)		
5–6 times per week	1103 (88.2)	148 (11.8)	1251 (11.3)		
7 times per week	5522 (87.7)	774 (12.3)	6296 (57.0)		
Missing	153 (89)	19 (11.1)	172 (1.6)		
Eating vegetables, *n* (%)				0.933	0.018 *
≤100g each day	1468 (83.7)	285 (16.3)	1753 (15.9)		
101–200g each day	4279 (88.2)	571 (11.8)	4850 (44.0)		
201–300g each day	2510 (87.7)	353 (12.3)	2863 (25.9)		
301–450g each day	690 (86.6)	107 (13.4)	797 (7.2)		
>450g each day	468 (88.5)	61 (11.5)	529 (4.8)		
Missing	213 (87.3)	31 (12.7)	244 (2.2)		
Eating fruits, n (%)				0.872	<0.001 ***
0 times per week	821 (81.6)	185 (18.4)	1006 (9.1)		
1–2 times per week	3570 (86.8)	544 (13.2)	4114 (37.3)		
3–4 times per week	2699 (88.2)	362 (11.8)	3061 (27.7)		
5–6 times per week	751 (86.4)	118 (13.6)	869 (7.9)		
Every day	1718 (90.3)	185 (9.7)	1903 (17.2)		
Missing	69 (83.1)	14 (16.9)	83 (0.8)		
Square dancing, *n* (%)				1.029	0.319
0 times per week	7147 (87.2)	1046 (12.8)	8193 (74.2)		
Occasionally	1114 (88.4)	146 (11.6)	1260 (11.4)		
1–2 times per week	546 (86.9)	82 (13.1)	628 (5.7)		
3–4 times per week	258 (82.7)	54 (17.3)	312 (2.8)		
≥5 times per week	341 (87.0)	51 (13.0)	392 (3.6)		
Missing	222 (88.5)	29 (11.6)	251 (2.3)		
Daily screen time, *n* (%)				1.016	0.104
Near 0 minutes	1178 (84.1)	223 (15.9)	1401 (12.7)		
>0 and <30 minutes	1118 (86.9)	169 (13.1)	1287 (11.7)		
30–60 minutes	1259 (89.6)	146 (10.4)	1405 (12.7)		
(1,2) hours	1335 (88.5)	173 (11.5)	1508 (13.7)		
(2,3) hours	1390 (88.5)	181 (11.5)	1571 (14.2)		
(3,4) hours	846 (88.9)	106 (11.1)	952 (8.6)		
(4,5) hours	625 (88.4)	82 (11.6)	707 (6.4)		
(5,6) hours	568 (87.1)	84 (12.9)	652 (5.9)		
(6,7) hours	290 (87.9)	40 (12.1)	330 (3.0)		
(7,8) hours	208 (85.3)	36 (14.8)	244 (2.2)		
(8,9) hours	227 (82.0)	50 (18.0)	277 (2.5)		
≥9 hours	367 (81.0)	86 (19.0)	453 (4.1)		
Missing	217 (87.2)	32 (12.9)	249 (2.3)		
Using electronic devices before sleep, *n* (%)				1.045	0.038 *
Never	1438 (84.6)	261 (15.4)	1699 (15.4)		
Rarely	1667 (90.2)	182 (9.8)	1849 (16.7)		
Sometimes	2147 (89.3)	257 (10.7)	2404 (21.8)		
Often	2331 (87.8)	325 (12.2)	2656 (24.1)		
Almost every day	1889 (84.0)	360 (16.0)	2249 (20.4)		
Missing	156 (87.2)	23 (12.9)	179 (1.6)		
Hypertension, *n* (%)					
Yes	1623 (83.4)	324 (16.6)	1947 (17.6)	1.475	<0.001 ***
No	7804 (88.1)	1056 (11.9)	8860 (80.3)	1.000	reference
Missing	201 (87.8)	28 (12.2)	229 (2.1)		
Diabetes, *n* (%)					
Yes	521 (78.5)	143 (21.5)	664 (6.0)	1.974	<0.001 ***
No	8910 (87.8)	1239 (12.2)	10,149 (92.0)	1.000	reference
Missing	197 (88.3)	26 (11.7)	223 (2.0)		
Hyperlipidemia, *n* (%)					
Yes	725 (81.4)	166 (18.6)	891 (8.1)	1.639	<0.001 ***
No	8707 (87.8)	1216 (12.2)	9923 (89.9)	1.000	reference
Missing	196 (88.3)	26 (11.7)	222 (2.0)		
Heart disease, *n* (%)					
Yes	556 (74.7)	188 (25.3)	744 (6.8)	2.513	<0.001 ***
No	8875 (88.1)	1194 (11.9)	10,069 (91.2)	1.000	reference
Missing	197 (88.3)	26 (11.7)	223 (2.0)		
Respiratory diseases, *n* (%)					
Yes	196 (75.1)	65 (24.9)	261 (2.4)	2.326	<0.001 ***
No	9235 (87.5)	1317 (12.5)	10,552 (95.6)	1.000	reference
Missing	197 (88.3)	26 (11.7)	223 (2.0)		
Stroke, *n* (%)					
Yes	129 (75.9)	41 (24.1)	170 (1.6)	2.205	<0.001 ***
No	9302 (87.4)	1341 (12.6)	10,643 (96.4)	1.000	reference
Missing	197 (88.3)	26 (11.7)	223 (2.0)		
Malignant tumor, *n* (%)					
Yes	34 (73.9)	12 (26.1)	46 (0.4)	2.421	0.009 *
No	9397 (87.3)	1370 (12.7)	10,767 (97.6)	1.000	reference
Missing	197 (88.3)	26 (11.7)	223 (2.0)		
Hyperuricemia, *n* (%)					
Yes	54 (78.3)	15 (21.7)	69 (0.6)	1.905	0.028 *
No	9377 (87.3)	1367 (12.7)	10,744 (97.4)	1.000	reference
Missing	197 (88.3)	26 (11.7)	223 (2.0)		
Musculoskeletal diseases, *n* (%)					
Yes	361 (75.7)	116 (24.3)	477 (4.3)	2.302	<0.001 ***
No	9070 (87.8)	1266 (12.2)	10,336 (93.7)	1.000	reference
Missing	197 (88.3)	26 (11.7)	223 (2.0)		

n, number; cOR was analyzed with one-way logistic regression; *: < 0.05, ***: < 0.001.

**Table 2:** Sleep Status of Participants.

	Frequency	Percentage (%)
Self-reported sleep quality		
Very good	1619	14.7
Good	3977	36.0
General	4032	36.5
Bad	1179	10.7
Very bad	229	2.1
Difficulty initiating sleep		
Never	4698	42.6
1–3 times per month	3622	32.8
1–2 times per week	1698	15.4
3–4 times per week	574	5.2
≥5 times per week	353	3.2
Missing	91	0.8
Difficulty maintaining sleep		
Never	5098	46.2
1–3 times per month	3349	30.4
1–2 times per week	1557	14.1
34 times per week	546	4.9
≥5 times per week	271	2.5
Missing	215	1.9
Awakening earlier		
Never	4923	44.6
1–3 times per month	3342	30.3
1–2 times per week	1629	14.7
3–4 times per week	560	5.1
≥5 times per week	394	3.6
Missing	188	1.7
Feeling sleepy during the day		
Never	4178	37.9
1–3 times per month	3401	30.8
1–2 times per week	2073	18.8
3–4 times per week	680	6.1
≥5 times per week	431	3.9
Missing	273	2.5
How long have you had one of above sleep problems?		
Never	4889	44.3
>0–<2 weeks	2498	22.6
2 weeks–1 month	1017	9.2
>1 month–3 months	592	5.4
>3 months–6 months	339	3.1
>6 months–12 months	392	3.6
>12 months	1050	9.5
Missing	259	2.3

**Table 3:** Associated Factors of Sleep Quality.

Associated Factors	Coefficient	*p* Value	aOR	95% CI
Sex				
Male (reference)	--	--	--	--
Female	0.583	<0.001 ***	1.792	(1.497, 2.144)
Marital status				
Married or remarried or cohabiting with boyfriend/girlfriend (reference)	--	--	--	--
Unmarried and without cohabiting with boyfriend/girlfriend	0.277	0.034 *	1.319	(1.022, 1.704)
Divorced or widowed	0.362	0.023 *	1.436	(1.052, 1.960)
Educational level				
College and above (reference)	--	--	--	--
Middle/high school	−0.290	0.003 **	0.748	(0.616, 0.908)
Primary school and below	0.067	0.580	1.069	(0.843, 1.357)
Family monthly income	−0.063	0.004 **	0.939	(0.900, 0.980)
Heart disease	0.391	0.002 **	1.478	(1.155, 1.891)
Musculoskeletal diseases	0.378	0.018 *	1.460	(1.067, 1.997)
Degree of concerning about their own health	0.107	0.016 *	1.113	(1.020, 1.214)
Self-reported health status	−0.714	<0.001 ***	0.490	(0.445, 0.539)
Drinking alcohol	0.183	<0.001 ***	1.201	(1.097, 1.315)
Having breakfast	−0.067	0.016 *	0.935	(0.886, 0.988)
Taking medication to help sleep	0.496	<0.001 ***	1.643	(1.498, 1.801)
Daily screen time	0.053	<0.001 ***	1.055	(1.027, 1.083)

*: < 0.05, **: < 0.01, ***: < 0.001.

## Data Availability

The data presented in this study are available on request from the corresponding author.

## References

[1] Cullen T., Thomas G., Wadley A.J., Myers T.D. (2019). The effects of a single night of complete and partial sleep deprivation on physical and cognitive performance: A Bayesian analysis. J. Sports Sci..

[2] Javaheri S., Barbe F., Campos-Rodriguez F., Dempsey J.A., Khayat R., Javaheri S., Malhotra A., Martinez-Garcia M.A., Mehra R., Pack A.I. (2017). Sleep Apnea. J. Am. Coll. Cardiol..

[3] St-Onge M.-P. (2017). Sleep-obesity relation: Underlying mechanisms and consequences for treatment. Obes. Rev..

[4] Steiger A., Pawlowski M. (2019). Depression and Sleep. Int. J. Mol. Sci..

[5] Shan Z., Majewski C., Xie M., Yan P., Guo Y., Bao W., Rong Y., Jackson C.L., Hu F.B., Liu L. (2015). Sleep Duration and Risk of Type 2 Diabetes: A Meta-analysis of Prospective Studies. Diabetes Care.

[6] McMakin D.L., Alfano C.A. (2015). Sleep and anxiety in late childhood and early adolescence. Curr. Opin. Psychiatry.

[7] Lo K., Keung V., Cheung C., Tam W., Lee A. (2019). Associations between Sleep Pattern and Quality and Cardiovascular Risk Factors among Macao School Students. Child. Obes..

[8] Wang L., Qin P., Zhao Y., Duan S., Zhang Q., Liu Y., Hu Y., Sun J. (2016). Prevalence and risk factors of poor sleep quality among Inner Mongolia Medical University students: A cross-sectional survey. Psychiatry Res..

[9] Dong X., Wang Y., Chen Y., Wang X., Zhu J., Wang N., Jiang Q., Fu C. (2018). Poor sleep quality and influencing factors among rural adults in Deqing, China. Sleep Breath..

[10] Wang Y., Li Y., Liu X., Liu R., Mao Z., Tu R., Zhang H., Zhang X., Qian X., Jiang J. (2019). Gender-specific prevalence of poor sleep quality and related factors in a Chinese rural population: The Henan Rural Cohort Study. Sleep Med..

[11] Wu W., Wang W., Dong Z., Xie Y., Gu Y., Zhang Y., Li M., Tan X. (2018). Sleep Quality and Its Associated Factors among Low-Income Adults in a Rural Area of China: A Population-Based Study. Int. J. Environ. Res. Public Health.

[12] Wang P., Song L., Wang K., Han X., Cong L., Wang Y., Zhang L., Yan Z., Tang S., Du Y. (2019). Prevalence and associated factors of poor sleep quality among Chinese older adults living in a rural area: A population-based study. Aging Clin. Exp. Res..

[13] Li J., Yao Y., Dong Q., Dong Y.-H., Liu J.-J., Yang L.-S., Huang F. (2013). Characterization and factors associated with sleep quality among rural elderly in China. Arch. Gerontol. Geriatr..

[14] Chen C. (2004). Guidelines for Prevention and Control of Overweight and Obesity in Chinese Adults. Acta Nutr. Sin..

[15] American Psychiatric Association (2014). Diagnostic and Statistical Manual of Mental Disorders.

[16] Shim J., Kang S.W. (2017). Behavioral Factors Related to Sleep Quality and Duration in Adults. J. Lifestyle Med..

[17] Grenier S., Payette M., Gunther B., Askari S., Desjardins F.F., Raymond B., Berbiche D. (2019). Association of age and gender with anxiety disorders in older adults: A systematic review and meta-analysis. Int. J. Geriatr. Psychiatry.

[18] Yang H.-C., Suh S., Kim H., Cho E.R., Lee S.K., Shin C. (2013). Testing bidirectional relationships between marital quality and sleep disturbances: A 4-year follow-up study in a Korean cohort. J. Psychosom. Res..

[19] Fok M., Stewart R., Besset A., Ritchie K., Prince M. (2009). Incidence and persistence of sleep complaints in a community older population. Int. J. Geriatr. Psychiatry.

[20] Gureje O., Oladeji B.D., Abiona T., Makanjuola V., Esan O. (2011). The Natural History of Insomnia in the Ibadan Study of Ageing. Sleep.

[21] Lao X., Liu X., Deng H.-B., Chan T.-C., Ho K.F., Wang F., Vermeulen R., Tam T., Wong M.C., Tse L. (2018). Sleep Quality, Sleep Duration, and the Risk of Coronary Heart Disease: A Prospective Cohort Study With 60,586 Adults. J. Clin. Sleep Med..

[22] Kwok C.S., Kontopantelis E., Kuligowski G., Gray M., Muhyaldeen A., Gale C.P., Peat G.M., Cleator J., Chew-Graham C., Loke Y.K. (2018). Self-Reported Sleep Duration and Quality and Cardiovascular Disease and Mortality: A Dose-Response Meta-Analysis. J. Am. Hear. Assoc..

[23] Chen Q., Hayman L.L., Shmerling R.H., Bean J.F., Leveille S.G. (2011). Characteristics of Chronic Pain Associated with Sleep Difficulty in Older Adults: The Maintenance of Balance, Independent Living, Intellect, and Zest in the Elderly (MOBILIZE) Boston Study. J. Am. Geriatr. Soc..

[24] Hartwell E.E., Bujarski S., Glasner-Edwards S., Ray L.A. (2015). The Association of Alcohol Severity and Sleep Quality in Problem Drinkers. Alcohol Alcohol..

[25] Ogilvie R.P., Lutsey P.L., Widome R., Laska M.N., Larson N., Neumark-Sztainer D. (2018). Sleep indices and eating behaviours in young adults: Findings from Project EAT. Public Health Nutr..

[26] Gwin J.A., Leidy H.J. (2018). Breakfast Consumption Augments Appetite, Eating Behavior, and Exploratory Markers of Sleep Quality Compared with Skipping Breakfast in Healthy Young Adults. Curr. Dev. Nutr..

[27] Mohammadbeigi A., Absari R., Valizadeh F., Saadati M., Sharifimoghadam S., Ahmadi A., Mokhtari M., Ansari H. (2016). Sleep Quality in Medical Students; the Impact of Over-Use of Mobile CellPhone and Social Networks. J. Res. Health Sci..

[28] Hale L., Guan S. (2015). Screen time and sleep among school-aged children and adolescents: A systematic literature review. Sleep Med. Rev..

[29] Tang J., Liao Y., Kelly B.C., Xie L., Xiang Y.-T., Qi C., Pan C., Hao W., Liu T., Zhang F. (2017). Gender and Regional Differences in Sleep Quality and Insomnia: A General Population-based Study in Hunan Province of China. Sci. Rep..

[30] Xiaoli Y., Xiaobing L., Xiaoxia A., Liang Z., Xianmei Z., Quanfu Y., Zongfen L., Liya Y. (2013). A Survey on Sleep Quality of the People Aged over 18 in Liaoning Province. Jian Kang Jiao Yu Yu Jian Kang Cu Jin.

[31] Chang S., Huijun W., Bing Z. (2020). Sleep status of adult residents aged 18-64 in 15 provinces (autonomous regions or municipalities) of China in 2015. Wei Sheng Yan Jiu.

